# Brain developmental trajectories in offspring of parents with severe mental illness

**DOI:** 10.1192/j.eurpsy.2025.134

**Published:** 2025-08-26

**Authors:** N. van Haren, DBSOS working group

**Affiliations:** 1 Child and adolescent psychiatry/psychology, Erasmus MC, Rotterdam; 2 Psychiatry, UMC Utrecht, Utrecht, Netherlands

## Abstract

**Abstract:**

Early diagnosis and intervention are essential for managing and improving long-term outcomes of severe mental illness, highlighting the need for reliable early biomarkers. This longitudinal study explores whether there are sex differences in the development of the brain during childhood and adolescence differs between offspring of parents with and without a diagnosis in the mood-psychosis spectrum.

We obtained 286 T1-weighted and diffusion weighted MRI scans of 184 offspring (aged 8–18 years at baseline) of at least one parent diagnosed with bipolar disorder (n=78) or schizophrenia (n=52) and offspring of parents without severe mental illness (n=54); 102 offspring underwent a follow-up scan (on average 3.9 years between scans). Global brain measures, regional cortical thickness and surface area, gyrification and sulcul morphology were computed. Anatomical brain networks were reconstructed into structural connectivity matrices. Network analysis was performed to investigate anatomical brain connectivity. Group comparisons and the interaction with age were analysed with (non)linear mixed-effects models. Explorative analyses will be done on the interaction with sex. To correct for multiple comparisons, we applied a Benjamini-Hochberg false discovery rate (FDR) correction (q=0.05).

A significant effect of age was found on most of the included brain features, with suggestive evidence for subtle deviations in trajectories in the cortical thickness and network metrics, but not in the gyrification index and sulcul morpholoy in offspring of parents with schizophrenia. Sex effects will be discussed during the meeting.

Our findings suggest the brain development in familial high-risk youngsters is associated with being at familial risk for schizophrenia.

**Disclosure of Interest:**

None Declared

